# Sediment Microbial Communities Influenced by Cool Hydrothermal Fluid Migration

**DOI:** 10.3389/fmicb.2018.01249

**Published:** 2018-06-13

**Authors:** Laura A. Zinke, Brandi Kiel Reese, James McManus, Charles G. Wheat, Beth N. Orcutt, Jan P. Amend

**Affiliations:** ^1^Marine and Environmental Biology Section, University of Southern California, Los Angeles, CA, United States; ^2^Department of Life Sciences, Texas A&M University – Corpus Christi, Corpus Christi, TX, United States; ^3^Department of Geosciences, The University of Akron, Akron, OH, United States; ^4^Bigelow Laboratory for Ocean Sciences, East Boothbay, ME, United States; ^5^Global Undersea Research Unit, University of Alaska Fairbanks, Moss Landing, CA, United States; ^6^Department of Earth Sciences, University of Southern California, Los Angeles, CA, United States

**Keywords:** cool hydrothermal systems, hydrothermal sediment, Dorado Outcrop, nitrogen, deep sea

## Abstract

Cool hydrothermal systems (CHSs) are prevalent across the seafloor and discharge fluid volumes that rival oceanic input from rivers, yet the microbial ecology of these systems are poorly constrained. The Dorado Outcrop on the ridge flank of the Cocos Plate in the northeastern tropical Pacific Ocean is the first confirmed CHS, discharging minimally altered <15°C fluid from the shallow lithosphere through diffuse venting and seepage. In this paper, we characterize the resident sediment microbial communities influenced by cool hydrothermal advection, which is evident from nitrate and oxygen concentrations. 16S rRNA gene sequencing revealed that Thaumarchaea, Proteobacteria, and Planctomycetes were the most abundant phyla in all sediments across the system regardless of influence from seepage. Members of the Thaumarchaeota (Marine Group I), Alphaproteobacteria (Rhodospirillales), Nitrospirae, Nitrospina, Acidobacteria, and Gemmatimonadetes were enriched in the sediments influenced by CHS advection. Of the various geochemical parameters investigated, nitrate concentrations correlated best with microbial community structure, indicating structuring based on seepage of nitrate-rich fluids. A comparison of microbial communities from hydrothermal sediments, seafloor basalts, and local seawater at Dorado Outcrop showed differences that highlight the distinct niche space in CHS. Sediment microbial communities from Dorado Outcrop differ from those at previously characterized, warmer CHS sediment, but are similar to deep-sea sediment habitats with surficial ferromanganese nodules, such as the Clarion Clipperton Zone. We conclude that cool hydrothermal venting at seafloor outcrops can alter the local sedimentary oxidation–reduction pathways, which in turn influences the microbial communities within the fluid discharge affected sediment.

## Introduction

Ridge flank hydrothermal systems are globally widespread and responsible for over two-thirds of marine hydrothermal heat flux (Stein and Stein, 1994). A significant amount of this flux is proposed to be through low temperature fluids at cool hydrothermal systems (CHSs). These systems bring cold seawater into the shallow lithosphere, circulate this fluid over short time scales of years to tens of years, and discharge cool (<20°C) fluid into the ocean through seafloor outcrops (Fisher et al., 2003; Wheat and Fisher, 2008; Wheat et al., 2017). More than 25 million seamounts and even more smaller basaltic outcrops that can facilitate CHS fluid flow are predicted to exist in the ocean (Wessel et al., 2010). Importantly, CHS systems discharge fluid volumes that rival oceanic input from rivers, with an estimated 10^14^ kg year^-1^ of fluid flowing through just the ∼15,000 largest seamounts (Harris et al., 2004). This fluid flux facilitated by CHS results in the removal of riverine phosphate and affects global budgets for other solutes (Wheat et al., 2003, 2017).

Although most discharge occurs directly through basaltic structures, a portion of the discharging fluid seeps upward through thin sediment. This fluid advection can elevate pore fluid nitrate and oxygen concentrations relative to sediment without fluid seepage (Wheat and Fisher, 2008; Wheat et al., 2017) and impact mineral composition (Bodeï et al., 2008). The delivery of oxidants from the crustal fluid makes high-energy electron acceptors, such as oxygen and nitrate, available for microbial metabolism at sediment depths where they would otherwise be depleted. Similar phenomenon was recently observed in sediment overlying the flank of the Mid-Atlantic Ridge, where diffusion of oxygen and nitrate into basal sediment ponded between crustal exposure stimulated a nitrogen-cycling microbial community (Reese et al., 2018).

Although the global significance of CHS to heat and chemical exchange has been demonstrated, there is a lack of understanding of how this fluid flux impacts the structure of sediment microbial communities or vice versa. Initial studies have demonstrated that oxic fluid flux changes the microbial community in basal sediment, but currently these studies have been performed at a limited numbers of sites, including much thicker sediment columns than at Dorado Outcrop (e.g., Reese et al., 2018) or at much greater temperature of source fluid (e.g., Huber et al., 2006). Hence, further characterization of CHS sites with shallow sediment cover (e.g., direct access of fluids to the overlying water) is necessary to determine how CHS fluids are impacting the associated sediment chemistry and thereby the microbial communities.

**FIGURE 1:**
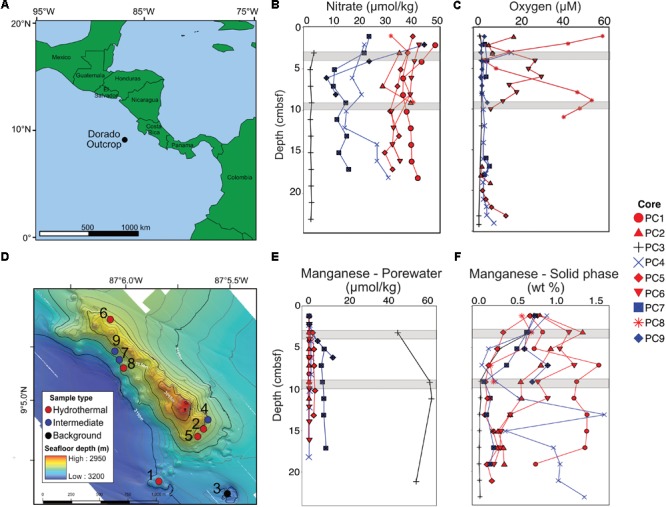
Overview of the Dorado Outcrop **(A)**, locations of sediment core samples **(D)**, and sediment geochemical profiles **(B,C,E,F)**. **(A)** The Dorado Outcrop is located in the Eastern Tropical Pacific Ocean on a 23-million-year-old ridge flank of the East Pacific Rise. **(B)** Sediment profiles of pore fluid nitrate concentrations with depth (centimeters below seafloor, cmbsf). **(C)** Dissolved oxygen concentrations of sediment push cores from Wheat et al. (2017). **(D)** Bathymetric map of Dorado Outcrop, which is approximately 2 km long and 0.5 km wide, rising approximately 150 m above the surrounding thickly sedimented seafloor. Push core locations are marked by circles, with the corresponding core designation labeled next to the circles. Adapted from Wheat et al. (2017). **(E)** Dissolved and **(F)** solid phase manganese profiles from sediment push cores. In **(B–F)**, sediment cores were classified based on nitrate profiles, which have been shown to demonstrate advective fluid flow from the ocean basement into the thin sediments on Dorado Outcrop (Wheat and Fisher, 2008). Symbol color represents hydrothermal (red), intermediate (blue), and background sediment (black) groups. Gray areas represent where samples were collected for 16S rRNA gene analysis.

**FIGURE 2:**
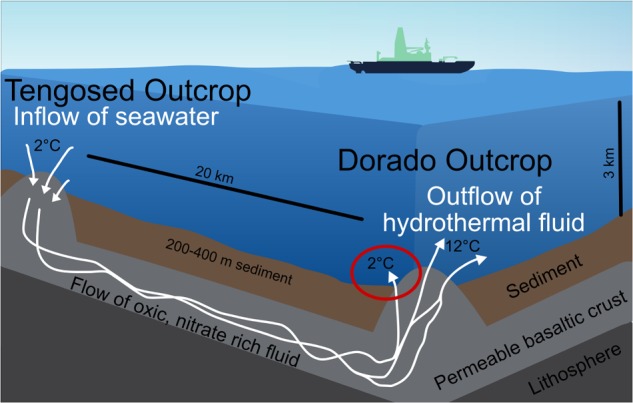
Schematic of the cool hydrothermal system (CHS) at Dorado Outcrop. Seawater enters the crust through recharging outcrops, such as Tengosed, 20 km distance from Dorado Outcrop. This cool seawater flows through the crust for <3 years before discharging at Dorado Outcrop (Wheat et al., 2017). The red circle denotes the focus of this study, the sediments on and near Dorado Outcrop which experience hydrothermal fluid discharge.

Here, we present a characterization of sediment microbial communities from the Dorado Outcrop (**Figures 1, 2**), located at approximately 3,000 m water depth on a 20- to 23-million-year-old region of the Cocos Plate (Wheat and Fisher, 2008). This outcrop is the first confirmed site of cool (<15°C) hydrothermal flow from a CHS (Hutnak et al., 2008; Wheat and Fisher, 2008), and recent investigations confirmed that venting fluids contain dissolved oxygen (<55 μM) and nitrate (<38 μM; Wheat et al., 2017). Sediment pore fluid profiles confirm the upward flux of oxygen and nitrate into basal sediment layers surrounding the outcrop, a signature of CHS fluid influence in the sediment (Wheat and Fisher, 2008; Wheat et al., 2017). Likewise, previous investigation of Dorado Outcrop sediment profiles of dissolved and solid phase manganese indicates oxidizing conditions in sediment influenced by CHS (i.e., less dissolved Mn and greater solid phase Mn), whereas background sediment not affected by CHS seepage had more reducing conditions (i.e., greater dissolved Mn and less solid phase Mn; Bodeï et al., 2008; Wheat and Fisher, 2008). In the background samples, solid manganese (i.e., manganese oxides) reduction by microorganisms resulted in the accumulation of manganese in pore fluids and depletion of solid phase manganese. The hydrothermal sediments did not show this pattern, indicating a more oxidizing sediment column and reflecting manganese concentrations similar to crustal fluid as opposed to a typical diagenetic pattern (Wheat and Fisher, 2008). A recent study of basalts on the outcrop did not indicate a clear signature of CHS flow on the structure of microbial communities on the exterior of the basalts, however (Lee et al., 2015). These prior characterizations make the Dorado Outcrop an ideal location to examine the potential influence of CHS seepage on sediment microbial communities.

The objectives of this study were to determine (1) the composition of microbial communities present in CHS sediments, (2) how hydrothermally affected sediment communities differed from those in nearby background sediments, seafloors basalts, and bottom seawater; (3) if geochemical changes associated with CHSs impact putative microbial metabolic potential at these sites; and (4) to determine how Dorado Outcrop compares to other deep-sea sites. We hypothesized that the elevated concentration of oxidized compounds (e.g., oxygen and nitrate) in hydrothermal sediments would alter the overall community composition relative to nearby sediment that was not affected by the seepage of hydrothermal fluids.

## Materials and Methods

### Site Description

The Dorado Outcrop is located in the Eastern Tropical Pacific Ocean (9°5′N, 87°5′W, **Figure 1A**) on a swath of seafloor derived from the East Pacific Rise (Fisher et al., 2003). The outcrop rises approximately 100–150 m above the surrounding seafloor, is 2 km long, and 0.5 km wide (**Figure 1D**) (Wheat et al., 2017). The sediment package surrounding the outcrop is approximately 200–400 m thick (Spinelli et al., 2006).

### Sample Collection

Sediment samples were collected from the Dorado Outcrop December 4–10, 2014 during cruise AT26-24 aboard the *R/V Atlantis*. Sediment was retrieved via push core (10–28 cm length) using the *DSV Alvin* during dive numbers 4777, 4780, 4782, and 4783 (**Figure 1D**). Cores for geochemistry and microbiology were taken adjacent to each other, with all related cores taken within half a meter from one other. These cores will be referred to hereafter as Push Core (PC) 1–9 (**Figure 1** and **Table 1**). Based on location and geochemical characterization described below, samples from PC1, 2, 5, 6, and 8 are collectively referred to as ‘hydrothermal’; samples from PC4, 7, and 9 as ‘intermediate’; and samples from PC3 as ‘background.’

**Table 1:** Characteristics of sediment samples from on and near Dorado Outcrop that were used for 16S rRNA gene sequencing (see **Figure 1** for more detail).

Core number	Core designation	Depth (cmbsf)	Oxygen (μM)	Nitrate + Nitrite (μM)	Solid phase Mn (wt %)
PC1	Hydrothermal	3–4	–	43.01	0.818
		9–10	–	37.31	1.252
PC2	Hydrothermal	3–4	5.9	34.68	1.324
		9–10	–	39.02	0.534
PC3	Background	3–4	1.1	1.65	0.679
		9–10	–	0.74	0.030
PC4	Intermediate	3–4	10.7	21.14*	0.293
		9–10	1.8	14.16	0.045
PC5	Hydrothermal	3–4	6.5	37.84	0.311
		9–10	–	31.11	0.085
PC6	Hydrothermal	3–4	26.9	40.47	1.149
		9–10	5.6	31.62	0.753
PC7	Intermediate	3–4	2.1	21.10	0.614
		9–10	2.5	13.98	0.066
PC8	Hydrothermal	3–4	14.6	38.38	0.668
		9–10	54.7	40.33	0.189
PC9	Intermediate	3–4	0.7	23.00	0.629
		9–10	3.5	10.09	0.682

*Core designation based on nitrate+nitrite concentrations, which were indicative of hydrothermal fluid flow through sediments (Wheat and Fisher, 2008). Oxygen values are from Wheat et al. (2017). A dash (–) indicates that the value was not measured for this sample. ^∗^Nitrate+nitrite value from 2 cmbsf depth.*

Once shipboard, cores were examined for cracks or seawater intrusion. Cores with no visible evidence of seawater intrusion were stored vertically at 4°C until pore fluid extraction or sediment sectioning could begin, usually within 12 h of sampling. Dissolved oxygen data were obtained by microsensor measurements through side ports of companion cores (Wheat et al., 2017). Pore fluids to measure dissolved nitrate and manganese were extracted with Rhizons (Rhizosphere Research Products), which were inserted through pre-drilled side ports of individual sediment cores (e.g., Ziebis et al., 2012). Samples for bulk manganese compositions were determined from 1-cm to 10-cm intervals that were selected at sea and refrigerated for further shore-based handling and analysis. Sediment samples for microbial analysis were taken from the center of the cores using sterile plastic syringes that had been cut off at one end, wrapped in foil, and autoclaved before use. Samples were collected in 6 cm intervals from each sediment core and immediately frozen at -70°C. Samples were shipped on dry ice to the University of Southern California and stored at -80°C until DNA extraction.

### Nitrate and Manganese Measurement

Dissolved nitrate and manganese were determined using colorimetric or ICP emission techniques, respectively, that were identical to those used to analyze discrete samples of discharging fluids (Wheat et al., 2017). Dithionite extractable manganese was determined ashore on freeze-dried sediment that was crushed with an agate mortar and pestle. The dithionite extractable manganese concentration was determined using a single step chemical leach that was designed to remove labile metals from sediment (Mehra and Jackson, 1960; Roy et al., 2013; Murray et al., 2013). Briefly, 0.25 g of sediment was heated at 60°C for 4 h in a buffered sodium dithionite solution with agitation via vortex every 15 min. Samples were then cooled before centrifuging at 4000 RPM for 5 min. The supernatant was decanted, diluted (1:20 with 18 MΩ water) and analyzed for manganese with a Perkin Elmer Atomic Absorption Spectrometer AAnalyst 700, or on an Agilent Technologies 700 series inductively coupled plasma optical emission spectrometer (ICP-OES). Dissolved and solid phase geochemical data are available from the Biological and Chemical Oceanography Data Management Office (BCO-DMO) database under project number 627844.

### DNA Extraction

DNA was extracted in triplicate from stored, frozen sediment (subsectioned further into 3–4 and 9–10 cm below seafloor, cmbsf). For each replicate, approximately 1 g of sediment was divided between two screw-cap 2-mL tubes. DNA was extracted in a UV-sterilized clean hood using the FastDNA SPIN Kit (MP Biomedicals, Santa Ana, CA, United States) using two reactions for each replicate. The two reactions for each replicate were combined during the SPIN filter step. Biological replicates were not combined. DNA was eluted in 50 μL of molecular biology grade sterile water. Blank extraction controls with no sample added were run alongside each extraction to verify sterility. Resulting extractions were quantified using the Qubit HS dsDNA Assay on a Qubit 2.0 Fluorometer (Life Technologies, Carlsbad, CA, United States) following manufacturer protocols.

### DNA Sequencing

A total of 54 samples (triplicates of two depths from nine push cores) and three extraction blanks were sequenced at the Molecular Research DNA Lab (Shallowater, TX, United States). The V4 region of the 16S rRNA gene was amplified using the Earth Microbiome Project universal 515F (5′-GTG CCA GCM GCC GCG GTA A-3′) and 806R (5′-GGA CTA CHV GGG TWT CTA AT-3′) primers (Caporaso et al., 2012). The forward primers included eight nucleotide barcodes. Libraries were created through PCR with HotStarTap Plus (Qiagen, Germantown, MD, United States) using the following protocol: 94°C for 3 min; 28 cycles of 94°C for 30 s, 53°C for 40 s, and 72°C for 1 min; and 72°C for 5 min. Amplified DNA was pooled in approximately equimolar concentrations and purified using Ampure XP beads (Beckman-Coulter, Indianapolis, IN, United States). Due to low initial DNA concentration, extraction blank libraries did not produce signal after the initial 28-cycle amplification, so the PCR was extended to 40 cycles prior to sequencing. DNA was sequenced on an Illumina MiSeq platform using Illumina TruSeq (Illumina, Inc., San Diego, CA, United States) chemistry with 2 × 250 base pair chemistry. Resulting sequence data were trimmed of barcodes and low-quality sequences using a quality cutoff of 25, and sequence read pairs were merged by the sequencing facility, resulting in an average sequence length of 299 base pairs.

### Sequence Analysis

Sequence primers were removed using Cutadapt (Martin, 2011). Sequence files containing all sequences were split into individual files using the split_libraries_fastq.py and split_sequence_file_on_sample_ids.py commands in QIIME (Caporaso et al., 2010). Merged sequences were processed in Divisive Amplicon Denoising Algorithm 2 (DADA2) v1.6 following the described protocols (Callahan et al., 2016a,b) implemented in R version 3.4.1 (R Core Team, 2016), and the following analyses were performed in DADA2 unless otherwise stated. Sequences primers were removed using Cutadapt (Martin, 2011). Sequence files containing all sequences were split into individual files using the split_libraries_fastq.py and split_sequence_file_on_sample_ids.py commands in QIIME (Caporaso et al., 2010), including a minimum quality score threshold of 25 for all sequences. These sequences were imported into DADA2, where they were further filtered and trimmed to a length of 240 base pairs following the suggested DADA2 workflow. Amplicon Sequence Variants (AVSs), which are analogous to 100% sequence similarity OTUs, were inferred from sequence data (Callahan et al., 2016b). Sequences representative of each ASV were chimera checked and chimeric ASV were removed. Sourcetracker was used to identify potential ASVs sourced from lab or DNA extraction kit contamination, and these ASVs were removed from samples before further downstream analyses (Knights et al., 2011). Taxonomy (phylum through genus levels) was assigned to ASVs using the Ribosomal Database Project’s naïve Bayesian classifier (Cole et al., 2009) with the Silva v128 database as the reference (Pruesse et al., 2007). Sequences from the current study were deposited into the National Center for Biotechnology Information (NCBI) Sequence Read Archive (SRA) database under project PRJNA433243.

Dorado Outcrop bottom seawater and seafloor basalt sequences collected in 2013 were included in sequence processing in order to assess potential contamination of sediment by seawater intrusion and similarities between CHS impacted sample types (i.e., basalt and sediment). Collection, extraction, and sequencing details are included in Lee et al. (2015). These samples were extracted using the same brand and type of extraction kit (MP Biomedicals FastDNA Spin Kit) and were sequenced with the same primer set (EMP 515F – 806R) at Molecular Research DNA Lab (Lee et al., 2015). These sequences are accessible at NCBI SRA project SRP063681.

Sequences from an oligotrophic sediment under the North Pacific Gyre (Clarion Clipperton Zone) were downloaded from NCBI SRA project SRP057408. These sequences were collected and sequenced as described previously (Shulse et al., 2017). DNA extraction and sequencing protocols were similar to those described here: DNA extraction was performed with the FastDNA Spin Kit, and sequencing was performed with the EMP 515F – 806R primer set on the Illumina MiSeq platform.

Dorado Outcrop sediment communities were compared to other sediments (Durbin and Teske, 2011; Tully and Heidelberg, 2016; Labonté et al., 2017; Reese et al., 2018) (**Supplementary Figure S9**) to determine how CHS sediments here are similar or different from other sites. These datasets were produced with different sequencing technologies, different primers, and in some cases contained drilling contaminants associated with deep biosphere samples, thus these comparisons are qualitative. Relative abundances of taxa were estimated from published data.

### Statistics

Statistical analyses between the 16S rRNA gene sequences (abundance and taxonomic assignment) and sediment geochemical data were performed using the phyloseq v1.22.3 (McMurdie and Holmes, 2013), DESeq2 v3.6 (Love et al., 2014), and Vegan v2.4.4 packages in R (Dixon, 2003). Plots were generated using ggplot2 (Wickham, 2011) and base R. Taxonomic assignment bar charts were generated in phyloseq using ggplots2. Relative abundances of data were determined by dividing the number of sequences for the ASV or taxon by the total number of quality controlled sequences for each sample. Ordinations (including NMDS, PCoA, and CCA) were generated using non-rarefied log transformed or rank abundance data (Callahan et al., 2016b) and the Bray–Curtis dissimilarity index (Bray and Curtis, 1957). Ordinations were plotted using ggplot2 v2.2.1 (Wickham, 2011). Differential abundance data and statistics were generated on ASVs using the DESeq2 package using the Wald test statistic and parametric fit (Love et al., 2014). Correlations between tested parameters and ASVs were considered significant when the *p*-value was less than 0.05. Additional statistical testing, including Student’s *t*-tests, linear regressions, and NP-MANOVA analyses were conducted in R using the vegan package (Dixon, 2003). Violin plots were made in R by agglomerating ASVs at the order level and plotting them based on abundance. PCoA analysis with weighted Unifrac was produced in QIIME (Caporaso et al., 2010). Alpha diversity was calculated in phyloseq using rarefied data (**Supplementary Table S5**).

## Results

### Sediment Geochemistry

Dissolved nitrate, oxygen, and manganese depth profiles differed among core classifications (**Figures 1B,C,E**). The background sediment core (PC3) had very low nitrate concentrations (**Figure 1B**). The hydrothermal cores had nitrate concentrations (∼30–40 μmol kg^-1^) similar to that measured in bottom seawater, a pattern previously interpreted as an indication of CHS advection (Wheat and Fisher, 2008). Nitrate concentrations in the three intermediate cores (6.51–44.31 μmol kg^-1^) were between those in the hydrothermal and background cores. The background core (PC3) was depleted in dissolved oxygen and ranged between 0 and 1.1 μmol kg^-1^ (**Figure 1C**). Two hydrothermal cores had measureable oxygen throughout most of the sediment column (PC8 and PC6). Some profiles also show an increase in oxygen concentration with increasing sediment depth near the sediment–basement interface (**Figure 1C**). The pore fluid manganese concentration was greater in the background sediment core (PC3) than in the hydrothermal and intermediate cores, where concentrations were typically below detection limit (**Figure 1E**). This pattern is consistent with prior observations where dissolved Mn is lower in sediment influenced by CHS seepage (Wheat and Fisher, 2008). Solid phase manganese concentrations in the cores varied between below detection limit (typically less than or equal to 0.03 wt %) and 1.7 wt % of dry sediment (**Figure 1F**). The background core showed solid phase manganese depletion in the first 10 cmbsf. Hydrothermal and intermediate core profiles did not show a clear pattern, but generally solid manganese decreased with depth.

### 16S rRNA Gene Sequencing, Clustering, and Taxonomic Assignment

Quality filtering of 4,659,316 paired-end sequences resulted in 4,459,234 sequences. The number of sequences per sample ranged from 6,786 to 127,268, with a mean of 78,232 ± 24,884 sequences (**Supplementary Table S1**). Clustering and chimera checking in DADA2 (Callahan et al., 2016a) produced 19,577 ASVs. Sediment communities were diverse, with no apparent pattern among samples (**Supplementary Table S5**).

Sourcetracker and PCA analysis showed there was little (<0.01%) taxonomic overlap between the ASVs from the sediment microbial communities with those from extraction blanks and bottom water (bottom water data from Lee et al., 2015, **Supplementary Figures S1, S2**). Clustering of triplicate sample sequence libraries using the Bray–Curtis dissimilarity index revealed consistency (clustering) of microbial community structure amongst replicates for most samples, with only two samples not clustering together (**Supplementary Figure S3**), indicating repeatable, quality sequencing per sample.

**FIGURE 3:**
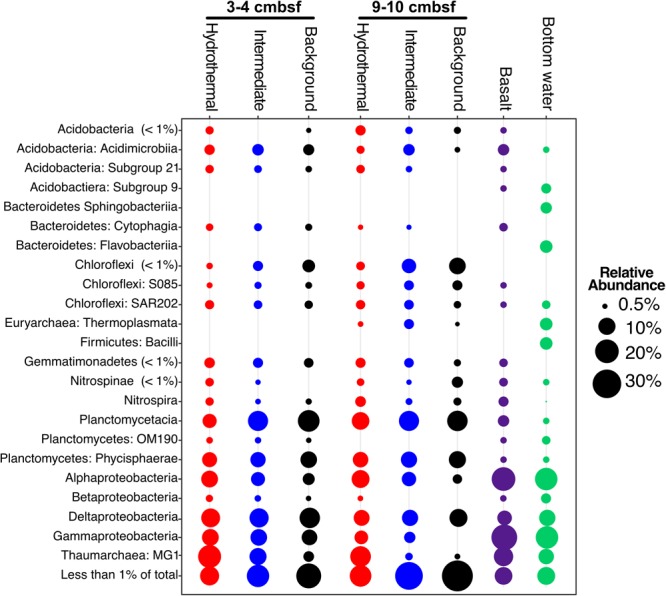
Taxonomic abundance of classes over 1% of the total community in Dorado Outcrop sediment, basalt, and bottom seawater samples based on 16S rRNA gene sequencing using the EMP primer pair (515F – 806R). Basalts and bottom seawater data from Lee et al. (2015).

The major phyla in all sediment samples on and near Dorado Outcrop were Proteobacteria (29.9 ± 6.1%), Planctomycetes (19.9 ± 4.5%), Thaumarchaeota (12.6 ± 7.7%), and Chloroflexi (7.8 ± 3.1%); an average of 6 ± 3% of sequences could not be assigned to a taxonomic group at the phylum level (**Figure 3** and **Supplementary Figure S4**). Within the Proteobacteria, the Delta class was the most abundant (12.8 ± 3% of total community), followed by the Alpha (8.5 ± 3.2%), and Gamma (7.7 ± 3.4%) classes (**Figure 3** and **Supplementary Figures S4–S6**). The Alphaproteobacteria were mostly within unclassified genera in the Rhodospirillaceae family (77.3% of Alphaproteobacteria), and the Gammaproteobacteria were mostly assigned as genera in the Xanthomonadales order (67.9% of Gammaproteobacteria) (**Supplementary Table S3**). The most abundant classified genera were the *Candidatus* Scalindua (9.1 ± 4.3%), the *Candidatus* Nitrosopumilales (5.0 ± 3.8%), the Urania-1B-19 marine sediment group genus within the Physcisphaereae family (2.4 ± 0.9%), Nitrospira (1.4 ± 1.5%), the H16 genus within the Desulfurellaceae family (Deltaproteobacteria) (1.3 ± 0.8%), the Pir4 lineage within the Planctomycetaceae (1.1 ± 0.5%), Nitrospina (0.8 ± 0.9%), *Candidatus* Omnitrophus (0.3 ± 0.2%), and *Nitrosomonas* (0.3 ± 0.2%) (**Supplementary Figure S5** and **Supplementary Table S4**).

**FIGURE 4:**
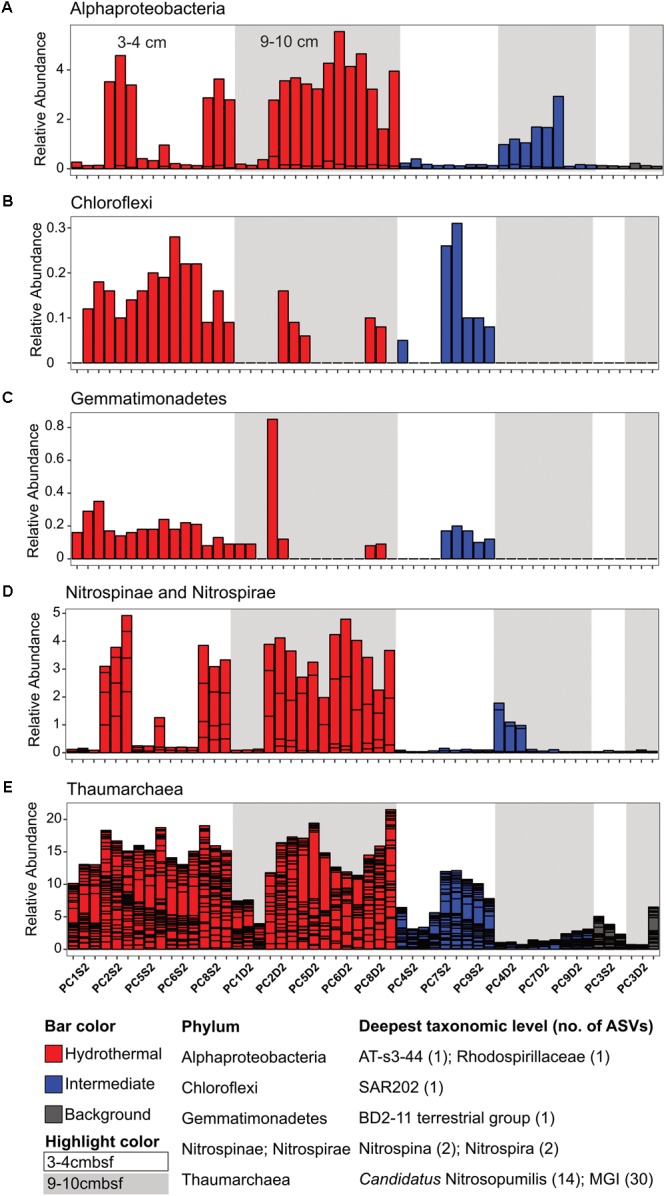
Select ASVs associated with **(A)** Alphaproteobacteria, **(B)** Chloroflexi, **(C)** Gemmatimonadetes, **(D)** Nitrospinae and Nitrospirae, and **(E)** Thaumarchaea which were more abundant in the hydrothermal and/or the hydrothermal plus intermediate samples. Bars are colored by sample, and divided by ASV abundance. Relative abundance is in percent of total sequences per sample. The deepest taxonomic level assigned are listed next to the phylum followed by the number of ASVs graphed.

**FIGURE 5:**
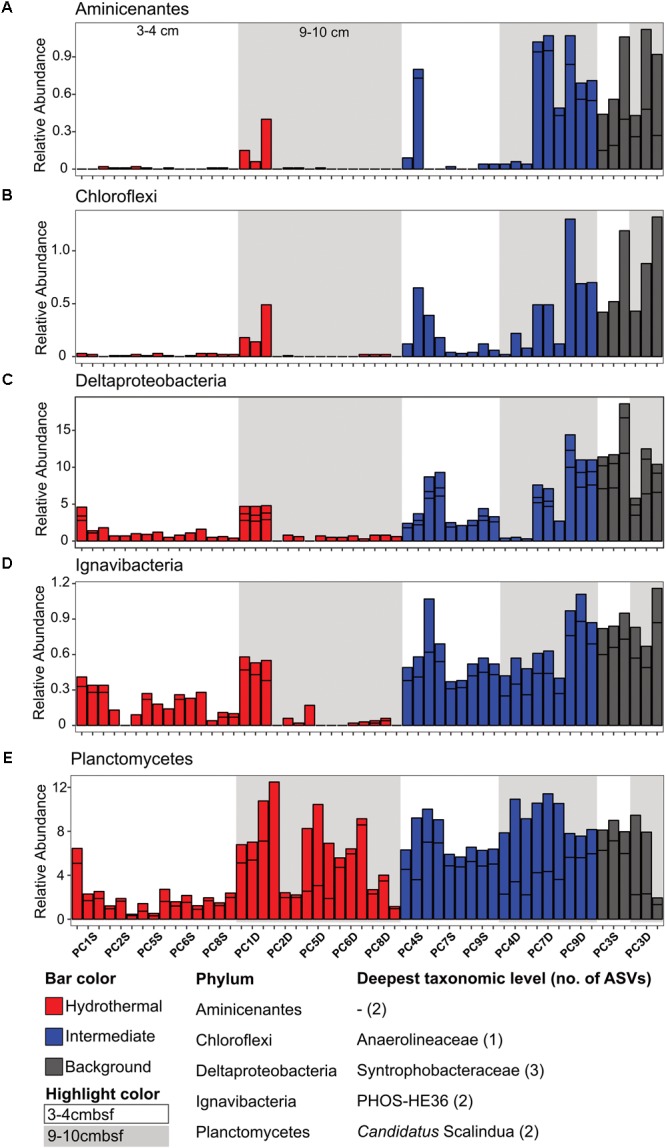
Select ASVs associated with **(A)** Aminicenantes, **(B)** Chloroflexi, **(C)** Deltaproteobacteria, **(D)** Ignavibacteria, and **(E)** Planctomycetes which were more abundant in the background and/or the background plus intermediate samples. Bars are colored by sample, and divided by ASV abundance. Relative abundance is in percent of total sequences per sample. The deepest taxonomic level assigned are listed next to the phylum followed by the number of ASVs graphed.

There were some notable differences in ASV distribution between sample types (**Figures 4, 5** and **Supplementary Figure S6**). Thaumarchaea assigned ASVs were notably more abundant in the HF compared to the BG samples (**Figure 4E**). Other ASVs enriched in hydrothermal sediment layers compared to the other sample types grouped within the Nitrospina (ASVs 82 and 92), Nitrospira (ASVs 12 and 23), Gemmatimonadetes class BD2-11 (ASV93), Alphaproteobacteria family Rhodospirillaceae (ASV 3), and SAR202 Chloroflexi (ASVs 235 and 402) (**Figure 4**). By contrast, ASVs which were more abundant in the background and/or intermediate samples were assigned as *Candidatus* Scalindua in the Planctomycetes (including ASVs 1 and 2), Ignavibacteria family PHOS-HE36 (ASV29), Deltaproteobacteria family Syntrophobacteraceae (ASV 38, 238, and 260), Aminicenantes ASVs (ASV87 and 232), and Chloroflexi family Anaerolineaceae (ASVs 47) (**Figure 5**). Of the most abundant ASVs, many ASVs (68 of the 100 most abundant) were either present in relatively similar abundances across samples (e.g., Actinobacteria and Gammaproteobacteria ASVs) or showed no apparent pattern (**Supplementary Figures S7, S8**). These observations were supported by differential abundance testing using DESeq2 on community composition data to determine which microbial taxa were significantly different among sample types (e.g., hydrothermal, intermediate, and background; **Supplementary Figure S6** and **Supplementary Table S2**). Student’s *t*-tests showed no significant differences in relative abundance of these taxa with depth in their classification (assuming a significance threshold of *p* < 0.05).

When samples were classified by nitrate concentrations, ASVs within 19 phyla were found to differ significantly (*p* < 0.05) between samples (**Supplementary Table S2**). Taxa that were positively associated with nitrate concentration included Thaumarchaeaota, Nitrosomonadales (within Betaproteobacteria), and Rhodospirillales (within Alphaproteobacteria). The ASVs that were significantly negatively associated with nitrate included those assigned to Anaerolineae and Dehalococcoides classes within the Chloroflexi phylum and Planctomycetes (**Supplementary Table S2**).

### Microbial Community Analysis

**FIGURE 6:**
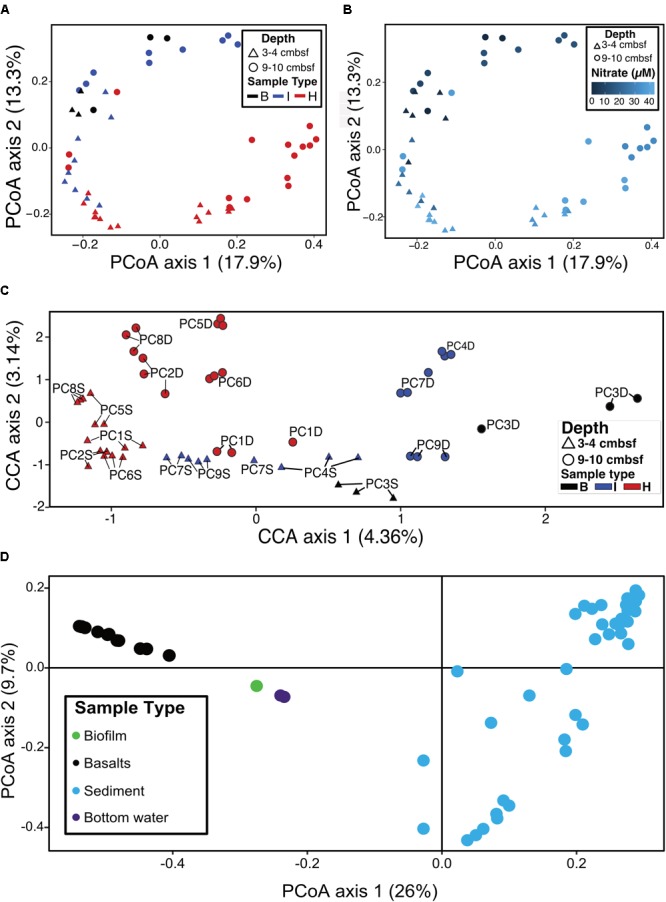
**(A)** Principal coordinates analysis (PCoA) of log transformed ASV abundance data showing clustering of samples by similarity using the Bray–Curtis dissimilarity index based on rank abundance, with points (samples) colored by sample type. **(B)** PCoA as in **(A)**, with color coded by nitrate concentration. **(C)** Canonical correspondence analysis (CCA) of samples based on Bray–Curtis dissimilarity index using log-transformed ASV frequencies, nitrate concentrations, and solid phase manganese concentrations. Labels show the core number (PC#), D indicates a deep sample (9–10 cmbsf) and S indicates a shallow sample (3–4 cmbsf). **(D)** PCoA of sediments (this study) and basalts, basaltic biofilm, and seawater samples (Lee et al., 2015).

Ordination analyses were used to determine if sample types (i.e., hydrothermal, intermediate, background) formed distinct groupings based on community composition. Principal coordinate analysis (PCoA) showed that background samples grouped separately from hydrothermal samples (**Figure 6A**). PCoA also showed that within sample types, some delineation of samples occurred. Sample groupings generally corresponded to nitrate concentrations when these were overlain onto the PCoA (**Figure 6B**). Canonical correspondence analysis (CCA) incorporated community composition, nitrate concentrations, and solid phase manganese content of samples into ordinations. As in the PCoA, hydrothermal communities were more similar to each other than they were to background communities (**Figure 6C**). Background communities from 9 to 10 cmbsf ordinated the furthest from other communities, and intermediate communities ordinated in between the background and hydrothermal communities. Within the sample types, samples grouped by depth, with the most pronounced trend in the hydrothermal samples (**Figure 6C**). An exception was sample PC1 9–10 cmbsf, a hydrothermal sample that ordinated most closely to intermediate 3–4 cmbsf samples.

Sediment samples were compared to seafloor basalts and bottom seawater described in Lee et al. (2015). Sediment communities were significantly different from basalt and seawater communities (*p* < 0.05), and ordination analyses showed grouping of the communities by sample types (**Figure 6D**). Notable differences among the communities were in the relative abundances of Bacteroidetes (1% and 2% of sediment and basalt, respectively, versus 9% of seawater), Chloroflexi (9% average of community between all sediments versus 2% average in basalts and seawater), and Planctomycetes (average 22% of sediment communities and only 6% and 4% of basalt and seawater communities, respectively) (**Figure 3** and **Supplementary Figure S4**).

**FIGURE 7:**
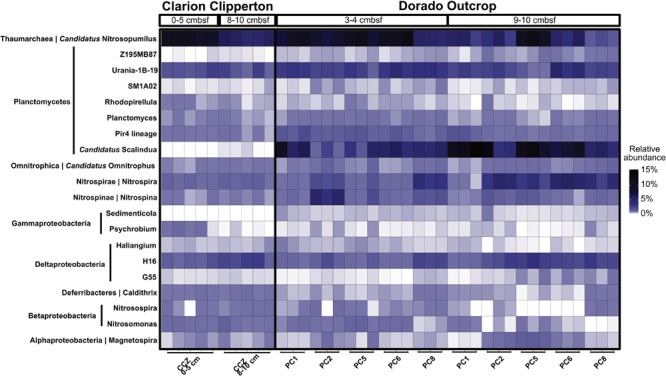
Relative abundance of genera in Clarion Clipperton Zone sediments and Dorado Outcrop hydrothermal sediments, at 0–5 cmbsf and 8–10 cmbsf for the Clarion Clipperton Zone, and 3–4 cmbsf and 9–10 cmbsf for the Dorado Outcrop sediments. Phylum is labeled to the left of the genus name along the *y*-axis.

Dorado Outcrop hydrothermal sediment communities were further compared to Clarion Clipperton Zone (Pacific Ocean) sediment communities, to examine how microbial communities within hydrothermally affected sediment from Dorado Outcrop compare to Pacific Ocean deep-sea sediment that is not affected by CHS but has similar concentrations of dissolved oxygen and nitrate. The most abundant classified genera (>0.1% of total community) were similar (**Figure 7**). Notably, *Candidatus* Nitrosopumilus, Planctomycetes lineages (including the Urania-1B-19, Rhodopirellula, Planctomyces, and Pir4 genera), the Deltaproteobacteria H16 genus, *Candidatus* Omnitrophus, Nitrospira, and Nitrospina were present in both sets of samples. Furthermore, the relative abundances of *Candidatus* Nitrosopumilus, Deltaproteobacteria H16, and *Candidatus* Omnitrophus were not significantly different (*t*-test, *p* > 0.05). However, some of the abundant genera were significantly enriched in Dorado Outcrop hydrothermal sediments (*t*-test, *p* < 0.05). These genera included Nitrospira, Nitrospina, and all of the Planctomycetes genera (**Figure 7**). Abundances of the *Candidatus* Scalindua showed the largest difference in abundances between the sites, with approximately 6.5% of Dorado Outcrop hydrothermal sequences assigned to the genus. In contrast, 0.02% of Clarion Clipperton Zone sequences were assigned to this genus. Overall, this comparison indicates that Dorado Outcrop hydrothermal sediments are similar to non-CHS deep-sea sediments, but some key differences (notably in the presence of *Candidatus* Scalindua) are apparent.

Dorado Outcrop communities shared many broad level taxonomic assignments with sediments examined from the South Pacific Gyre (Durbin and Teske, 2011; Tully and Heidelberg, 2016) (**Supplementary Figure S9**). The MGI Thaumarchaea were abundant in both sediments, as were Planctomycetes and Proteobacteria. Juan de Fuca (JdF) sediments contained higher percentages of Chloroflexi than seen here (**Supplementary Figure S9**), most of which were Dehalococcoides (Labonté et al., 2017), which were not abundant here (**Supplementary Figure S7**). North Pond communities contained more Cyanobacteria than any other sample, as well as Firmicutes (**Supplementary Figure S9**) (Reese et al., 2018) .

## Discussion

### CHS-Impacted Sediment Microbial Communities

Whereas many hot hydrothermal sediment microbial communities have been described in detail (Moyer et al., 1995; Teske et al., 2002; Cerqueira et al., 2015; Dowell et al., 2016), sediment communities impacted by discharge of cool, oxygen- and nitrate-containing basaltic formation fluids are less well understood. To determine how these communities evolve in response to fluid migration, we need to first understand microbial community structures in “typical” background (oligotrophic) sediment, in bottom seawater, in the fluids that migrate through ocean crust and discharge at outcrops, and on the basaltic rocks that the fluids are exposed to.

In this study, we determined that background and CHS-influenced sediment at the Dorado Outcrop (the first confirmed site of significant CHS discharge (Wheat et al., 2017) are dominated by the many of the same taxa (**Figure 3** and **Supplementary Figure S7**), but within this overall similarity, some taxa were correlated with CHS influence (**Figures 4, 5**). Some of these taxonomic groups (the genera Nitrospina, Nitrospira, and *Candidatus* Nitrosopumilus) are known to be involved in oxic nitrogen cycling processes (Könneke et al., 2005; Lücker et al., 2010, 2013; Walker et al., 2010; Rosenberg et al., 2014), suggesting that the advection of nitrate and/or oxygen is a strong driver favoring the stimulation of microbial groups that can perform such metabolic processes.

Dorado Outcrop sediments exhibit community compositions similar to those in other deep-sea sediments (**Supplementary Figure S9**). Dominant taxa in the top 10 cmbsf of sediments from the Clarion Clipperton Zone in the Eastern North Pacific were identified as Gammaproteobacteria, Alphaproteobacteria (specifically Rhodospirillaceae), Deltaproteobacteria, Planctomycetes (Phycispharae), and Thaumarchaea (including *Candidatus* Nitrosopumilus) (Shulse et al., 2017). These taxa were also among the most abundant taxa in hydrothermal sediments at Dorado, and analyses revealed similarities on the genus level (**Figure 7**), and often these community members were of similar relative abundances. Interestingly, *Candidatus* Scalindua was significantly enriched in Dorado Outcrop sediments, as were several other genera associated with nitrogen cycling (Nitrospira and Nitrospina). However, *Candidatus* Scalindua has been observed in clone libraries throughout marine sediments (Penton et al., 2006), including deep-sea sediments in the South China Sea (Hong et al., 2011).

In another example, microbial communities in shallow oligotrophic sediment of the South Pacific Gyre (Durbin and Teske, 2011) display similarities to those in Dorado sediment. Namely, in South Pacific Gyre (SPG) oxic zones, Planctomycetes (including *Candidatus* Scalindua), Chloroflexi (SAR-202, Dehalococcoides, Anaerolineae), Alphaproteobacteria (Rhodospirillales), Gammaproteobacteria, and Gemmatimonadetes are present, and the archaeal community was dominated by MG-1 Archaea (Durbin and Teske, 2011), which is similar to the oxic communities here. With increasing depth in the SPG sediment, oxygen and nitrate concentrations decreased, and the relative abundances of Chloroflexi (specifically Dehalococcoides and Anaerolineae) and Planctomycetes increased; this pattern is similar to that observed in Dorado Outcrop sediment (**Figure 4** and **Supplementary Figure S6**). Another striking similarity between Dorado Outcrop and SPG sediments was the disappearance of MG-1 Thaumarchaea in the oxygen- and nitrate-depleted SPG sediments. We observed a similar trend in Dorado Outcrop sediment. MG-1 sequences were still present in the anoxic background sediments, but at a much lower abundance compared to the nitrate-rich hydrothermal sediments.

Another example of this is the “North Pond” site on the western flank of the Mid-Atlantic Ridge has similar circulation of oxygen-enriched fluids within basement underneath the sediment (Ziebis et al., 2012; Orcutt et al., 2013). The sediment temperatures were similar at these two locations, and both had notable abundances of Alpha-, Beta-, and Gammaproteobacteria (Reese et al., 2018). These classes were abundant in the Dorado Outcrop sediment communities, though lower taxonomic levels were less similar. For instance, North Pond contained abundant sequences from the genera *Brevundimonas* (Alphaproteobacteria), *Achromobacter* and *Delftia* (Betaproteobacteria), and *Pseudomonas* (Gammaproteobacteria). Dorado Outcrop sediments did not contain significant portions (more than 1% abundance) of these taxa. Additionally, numerous diatom chloroplast sequences were preserved at North Pond, which were not found here. However, North Pond sediment microbial communities correlated with nitrate concentrations (Reese et al., 2018), as found in Dorado Outcrop sediment (**Figure 6**).

Overall, these results indicate that Dorado Outcrop CHS hydrothermal sediments contain similar microbial communities to other deep-sea (non-hydrothermal) sediments, with notable enrichment in groups that may be involved in nitrogen cycling (i.e., Nitrospira, Nitrospina, and *Candidatus* Nitrosopumilus). Additionally, Dorado Outcrop contained large relative abundances of the anammox bacteria *Candidatus* Scalindua. This is notable because previous characterizations of hydrothermally impacted microbial communities, even relatively cool ones, have shown distinct hydrothermal communities that are functionally and taxonomically distinct from other seafloor sediments (Urich et al., 2013; Cerqueira et al., 2015). However, here we see that CHS sediments are impacted by hydrothermal activity in the abundance of some taxonomic groups, but overall are similar to other oxic and nitrate-rich sediments. CHS microbial communities respond to CHS-related nitrogen fluxes from the subseafloor. Since potentially millions of seamounts facilitate CHS (Fisher and Wheat, 2010), the nitrogen cycle in these systems deserves further characterization.

By contrast, microbial communities in Dorado Outcrop sediment are less similar to those in warmer sediment at another outcrop system on eastern flank of the Juan de Fuca Ridge. Like Dorado sediments, outcrop sediments in the Juan de Fuca system are influenced by migration of basement fluids (Wheat and Mottl, 2000; Wheat et al., 2013). However, critical differences between these systems include temperature (up to 64°C at Juan de Fuca, Wheat and Mottl, 2000), system age (∼3.5 million years versus ∼20 million years old at Dorado), and fluid chemistry (anoxic and nitrate-depleted vs. oxic and nitrate-replete; Wheat and Mottl, 2000). These differences are reflected in the microbial community composition. Sediment from the Baby Bare outcrop, a site of hydrothermal fluid discharge, contained microbial communities dominated by Epsilonproteobacteria and Aminicenantes (OP8; Huber et al., 2006), which is not the case in Dorado Outcrop sediment (**Figure 3**). Likewise, different genera within the Chloroflexi and Deltaproteobacteria are present at the two sites (Huber et al., 2006). Furthermore, deeper anoxic sediments from around the Grizzly Bare Outcrop were heavily dominated by Dehaloccoides (Chloroflexi), Atribacteria, and Actinobacteria (Labonté et al., 2017), which do not dominate in Dorado hydrothermal sediments. The flux of oxygen and nitrate into Dorado Outcrop sediments, versus the anoxic fluid flux through the Juan de Fuca system, likely explains these divergent community compositions. Many of the taxa that are present in Dorado Outcrop sediments, but not Juan de Fuca, are associated with cool, oxic sediments, and the increased oxygen and nitrate concentrations at Dorado select for these communities. In contrast, the warmer, anoxic fluid flux at Juan de Fuca selected for the putatively anaerobic communities observed there.

Sediments from Dorado Outcrop contained microbial communities that were also distinct from nearby (within a ∼2 km^2^ area) seafloor-exposed basalts and bottom seawater (**Figures 3, 6D**), indicating that substrate plays an important role in the selection of these communities. This agrees with numerous other studies which have demonstrated that solid mineral substrates select for microbes capable of respiring these materials, and geochemistry determines the community composition (Templeton et al., 2005; Smith et al., 2011; Henri et al., 2016; Sudek et al., 2017). For instance, iron- and manganese-oxidizing bacteria are ubiquitous on marine basalts from active vents, and can be enriched on minerals found in basalts (Templeton et al., 2005; Henri et al., 2016). Inactive sulfide chimneys from other vent systems contained increased abundances of Alpha-, Beta-, Delta-, and Gammaproteobacteria relative to their active counterparts, and MGI Thaumarchaea were also overrepresented on the cool, inactive rocks. Dorado Outcrop basalts resemble the inactive sulfides by having abundant MGI Thaumarchaea, various Proteobacteria, and sulfur-oxidizing *Thioprofundum lithotrophicum* residents. The presence of MGI in both basalts and sediments could point to similarities between CHS sediment and basalt colonization at Dorado Outcrop, and both the sediment and basalts are communities that are distinct from their hotter counterparts. However, the enrichment of some taxa (e.g., *Candidatus* Scalindua) in sediments versus others in basalt (e.g., *Thioprofundum lithotrophicum*) demonstrate that despite common location and CHS influence, CHS basaltic and sediment biospheres are under distinct geochemical controls.

In total, sediment chemistry on Dorado Outcrop is driven by CHS conditions, and CHS associated chemistry impacts community structure and succession. Despite the differences between CHS sediments at Dorado and non-hydrothermal sites (notably active flux of crustal fluid through sediments versus diffusion controlled systems), the hydrothermal sediment communities on Dorado Outcrop are similar to other non-hydrothermal but oxic deep-sea sediments from the Pacific.

### Venting Causes Changes in Putative Microbial Metabolic Potential

Sediment microbial community succession is often driven by geochemical changes associated with reduction of terminal electron acceptors (Nealson, 1997). Our study from Dorado Outcrop expands upon this general paradigm to emphasize the importance of electron acceptor advection and diffusion from basement into sediment. At Dorado Outcrop, sediments that are influenced by CHS fluid advection processes have higher concentrations of oxygen and nitrate and lower dissolved manganese compared to background sediments (**Figure 1**), indicating upward delivery of electron acceptors and oxidizing conditions (Wheat and Fisher, 2008). Furthermore, statistical analysis of sediment microbial community profiles with geochemical parameters indicates that nitrate and oxygen exert a significant selective force on the Dorado Outcrop CHS-influenced sediment microbial community structure (**Figure 6**). Nitrogen respiration has increasingly been recognized as an important metabolism in oligotrophic deep sea sediments (Orcutt et al., 2013; Wankel et al., 2015; Reese et al., 2018). At Dorado Outcrop, taxonomic analyses of resident microbial communities revealed lineages in CHS sediments related to known nitrogen cycling organisms. However, the inference of function from taxonomy remains tenuous due to their capability to perform multiple functions and to processes like horizontal gene transfer, which is extensive in prokaryotes (Koonin et al., 2001). We recommend these metabolic functions as putative or hypothetical functions rather than absolute functions, and here we will focus on genera with cultured representatives that have been previously identified to have metabolisms related to nitrogen cycling, which have been found to be typically conserved phylogenetically (Martiny et al., 2013).

Hydrothermal samples contained significantly larger percentages of *Candidatus* Nitrosopumilus*-*related sequences than did intermediate and background, especially 9–10 cmbsf. Cultured representatives of these Thaumarchaea are known to aerobically oxidize ammonium to nitrite (Könneke et al., 2005; Walker et al., 2010), although recent studies have revealed some Thaumarchaea may be heterotrophic or mixotrophic (Swan et al., 2014), utilize urea in respiration (Alonso-Sáez et al., 2012), or anaerobically oxidize ammonium to nitrite (Jørgensen et al., 2012). Sequences also were assigned as Nitrosospira, a genus of Betaproteobacteria known to oxidize ammonium to nitrite (Prosser et al., 2014), including one ASV, which was among the most abundant ASVs here (**Supplementary Figure S5**). ASVs classified within the Nitrospira and Nitrospina genera were found in these sediments as well, pointing to aerobic oxidation of nitrite to nitrate (Lücker et al., 2010, 2013). In addition to the advection of nitrate rich hydrothermal fluid providing elevated nitrate, some nitrate might be produced *in situ* through the action of putative ammonium and nitrite oxidizers. In contrast, the relative abundance *Candidatus* Scalindua-related sequences in the intermediate and background sediments could indicate anaerobic ammonium oxidation (anammox) is a critical nitrogen cycling process in some sediments on and near Dorado Outcrop. This notion is in accordance with findings in other anoxic or suboxic sediments, which found that anammox can account for up to 80% of dinitrogen production (Devol, 2015).

## Conclusion

This study characterized microbial communities in ridge flank sediments associated with active cool hydrothermal discharge. Dissolved oxygen and nitrate in crustal fluid that seep through thin sediment establish geochemical conditions, especially in deeper sediments, that favored microbial communities different from those in background sediment. CHS-influenced sediment communities were diverse, and contained *Thaumarchaea, Proteobacteria, Planctomycetes*, and *Chloroflexi* related sequences. These communities were similar to those from other oxic cold surficial marine sediments, and included large percentages of taxa related to known aerobic nitrogen cycling organisms.

## Author Contributions

LZ, BKR, and JA designed the experiments. LZ, JM, CW, and BO collected samples. BO collected oxygen data. JM and CW analyzed geochemistry. LZ performed all molecular biology lab work and data analysis. All authors contributed to the writing and editing of the manuscript.

## Conflict of Interest Statement

The authors declare that the research was conducted in the absence of any commercial or financial relationships that could be construed as a potential conflict of interest.

## Supplementary Material

FIGURE S1 Taxonomic assignment at the family level of sequences from extraction blanks. These taxa were not found or were present in extremely low abundances in sediment samples, and when considered with sourcetracker analysis results, indicated a low level of contamination of samples during the extraction and sequencing process.

FIGURE S2 Principal coordinate analysis (PCoA) based on weighted UniFrac analysis of all sediment sample replicates (in red, blue, and black) before sourcetracker with extraction/sequencing blanks (green), as well as the seawater samples (purple). The closer the points are positioned to each other, the more similar the samples are. The further away the samples are from another, the less similar the samples are. Extraction/sequencing blanks are clustering away from all environmental samples.

FIGURE S3 Cluster diagram of all samples and replicates based on Bray–Curtis dissimilarity. Replicates are color coded. Shallow samples are from 3 to 4 cm below seafloor. Deep samples are from 9–10 cm below seafloor. Samples more closely positioned and sharing nodes are more similar.

FIGURE S4 Class level assignments of major groups in sediments on Dorado Outcrop, based on averages of triplicate samples. Thaumarchaea, Planctomycetes, and Proteobacteria classes dominate in the hydrothermal samples.

FIGURE S5 Relative abundances of genera at Dorado Outcrop. The *x*-axis includes all samples, with each replicate individually shown. The *y*-axis shows all assigned genera over 0.15% abundance. *Candidatus* Scalindua and *Candidatus* Nitrosopumilus are abundant in hydrothermal samples.

FIGURE S6 Violin plots of percentages of specific taxa separated by sample type (B, background; I, intermediate; H, hydrothermal) and depth (color coded as blue for 3–4 cmbsf and black for 9–10 cmbsf). On the *y*-axis, the violin plots display the distribution of relative percentages of the selected taxa in each sample type. **(a–f)** Shows taxa whose relative percentages were increased in hydrothermal sediments, relative to background or intermediate samples. **(g–l)** Shows taxa whose relative percentages were decreased in hydrothermal sediments.

FIGURE S7 The abundance of the top 100 ASVs and their taxonomic assignments which showed no distinct pattern between sample types, for Acidobacteria and Actinobacteria **(A)**; Bacteroidetes, Chloroflexi, Gemmatimonadetes, Euryarchaeota, and Ignavibacteria **(B)**; Nitrospinae, Nitrospirae, and Planctomycetes **(C)**; Alphaproteobacteria and Deltaproteobacteria **(D)**; Gammaproteobacteria **(E)**; and Thaumarchaea **(F)**. Relative abundance is in percent of total sequences per sample. The taxonomic levels listed are phylum followed by the most specific taxonomic level assigned (c, class; o, order; f, family; g, genus) and the taxon name. ASV114 could not be assigned beyond the phylum level.

FIGURE S8 Combined relative abundance of the top 300 ASVs (which included all ASVs considered in **Figures 4, 5**) as a proportion of the total community. The top 300 ASVs represented over half of the sequences in most samples, and included all ASVs over 1% abundance.

FIGURE S9 Comparison of Dorado Outcrop communities to communities from other deep sea sediments. Taxa are listed at the phylum level, except for the Proteobacteria, which are listed at the class level. Durbin and Teske separated Archaea and Bacteria clone library percentages, so the Thaumarchaea (MGI), which were 100% of the clones, are displayed as a large rounded square, and all Bacteria clones are percentages of only the Bacteria community.

TABLE S1 Sequence statistics during processing in DADA2.

TABLE S2 ASVs significantly correlated with nitrate or pore fluid manganese concentrations.

TABLE S3 Proportion of samples assigned at the order level in samples.

TABLE S4 Taxonomic assignments and abundances of top 100 most abundant ASVs, which were presenting in **Supplementary Figure S5**.

TABLE S5 Alpha diversity measures for each replicate.
