# Supplementary figures and images for: Sediment Microbial Communities Influenced by Cool Hydrothermal Fluid Migration
Source: Front Microbiol. 2018 Jun 13;9:1249. doi: 10.3389/fmicb.2018.01249 (PMC6008377; doi:10.3389/fmicb.2018.01249)

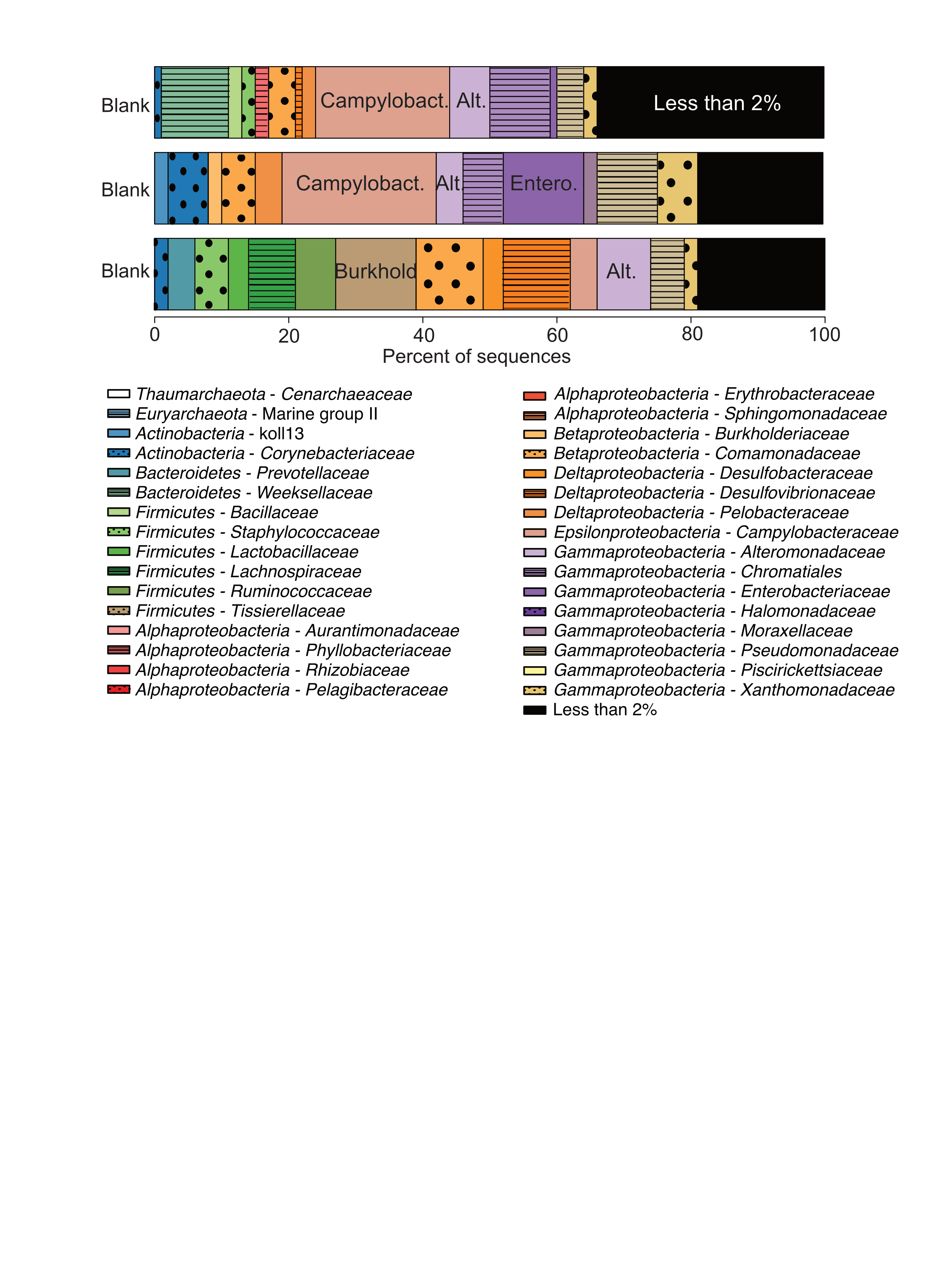

Supplement: FIGURE S1 — Taxonomic assignment at the family level of sequences from extraction blanks. These taxa were not found or were present in extremely low abundances in sediment samples, and when considered with sourcetracker analysis results, indicated a low level of contamination of samples during the extraction and sequencing process. [file Image_1.TIFF]

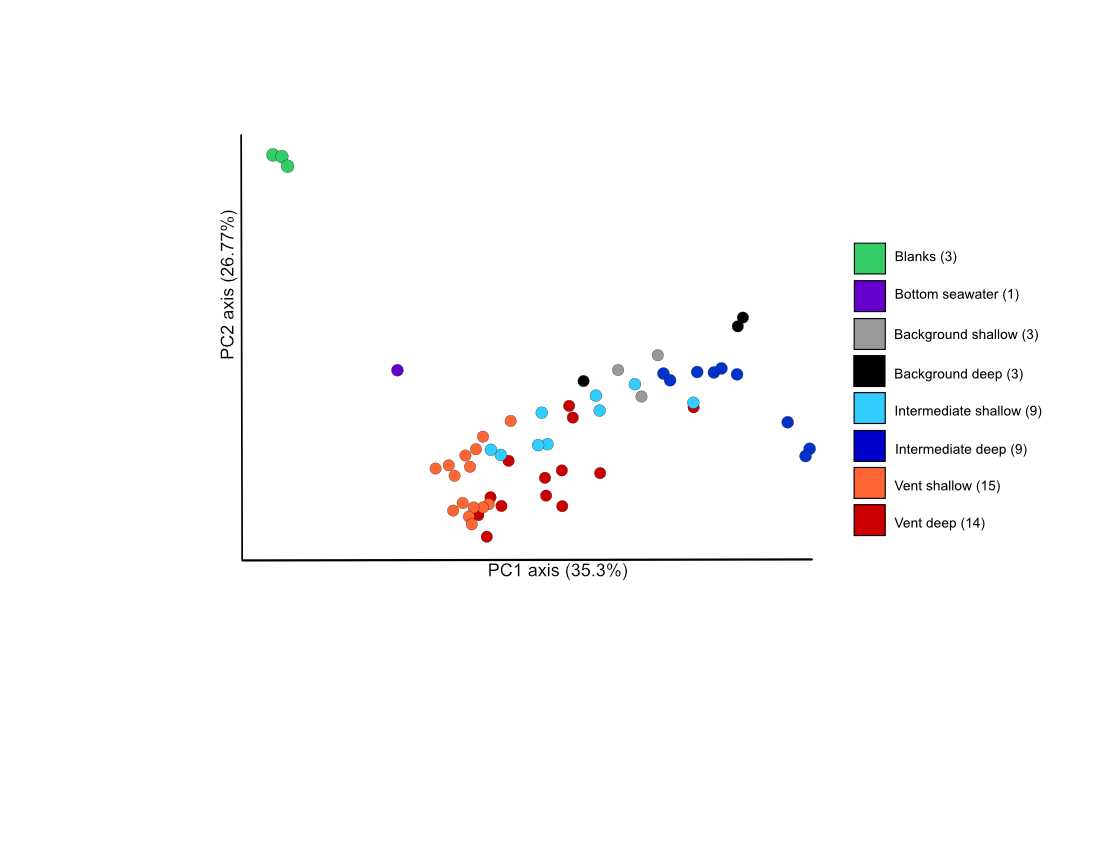

Supplement: FIGURE S2 — Principal coordinate analysis (PCoA) based on weighted UniFrac analysis of all sediment sample replicates (in red, blue, and black) before sourcetracker with extraction/sequencing blanks (green), as well as the seawater samples (purple). The closer the points are positioned to each other, the more similar the samples are. The further away the samples are from another, the less similar the samples are. Extraction/sequencing blanks are clustering away from all environmental samples. [file Image_2.TIFF]

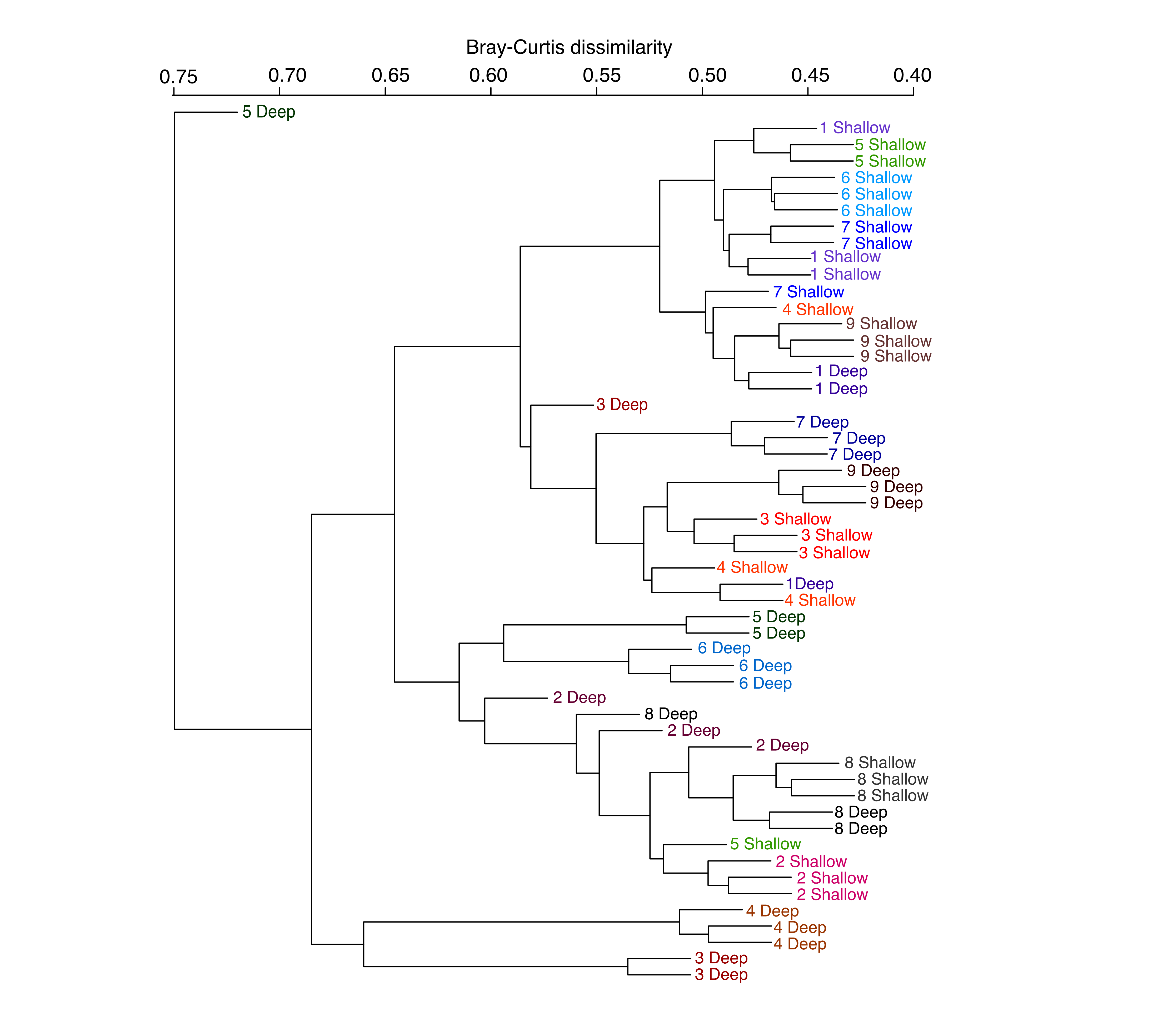

Supplement: FIGURE S3 — Cluster diagram of all samples and replicates based on Bray–Curtis dissimilarity. Replicates are color coded. Shallow samples are from 3 to 4 cm below seafloor. Deep samples are from 9–10 cm below seafloor. Samples more closely positioned and sharing nodes are more similar. [file Image_3.JPEG]

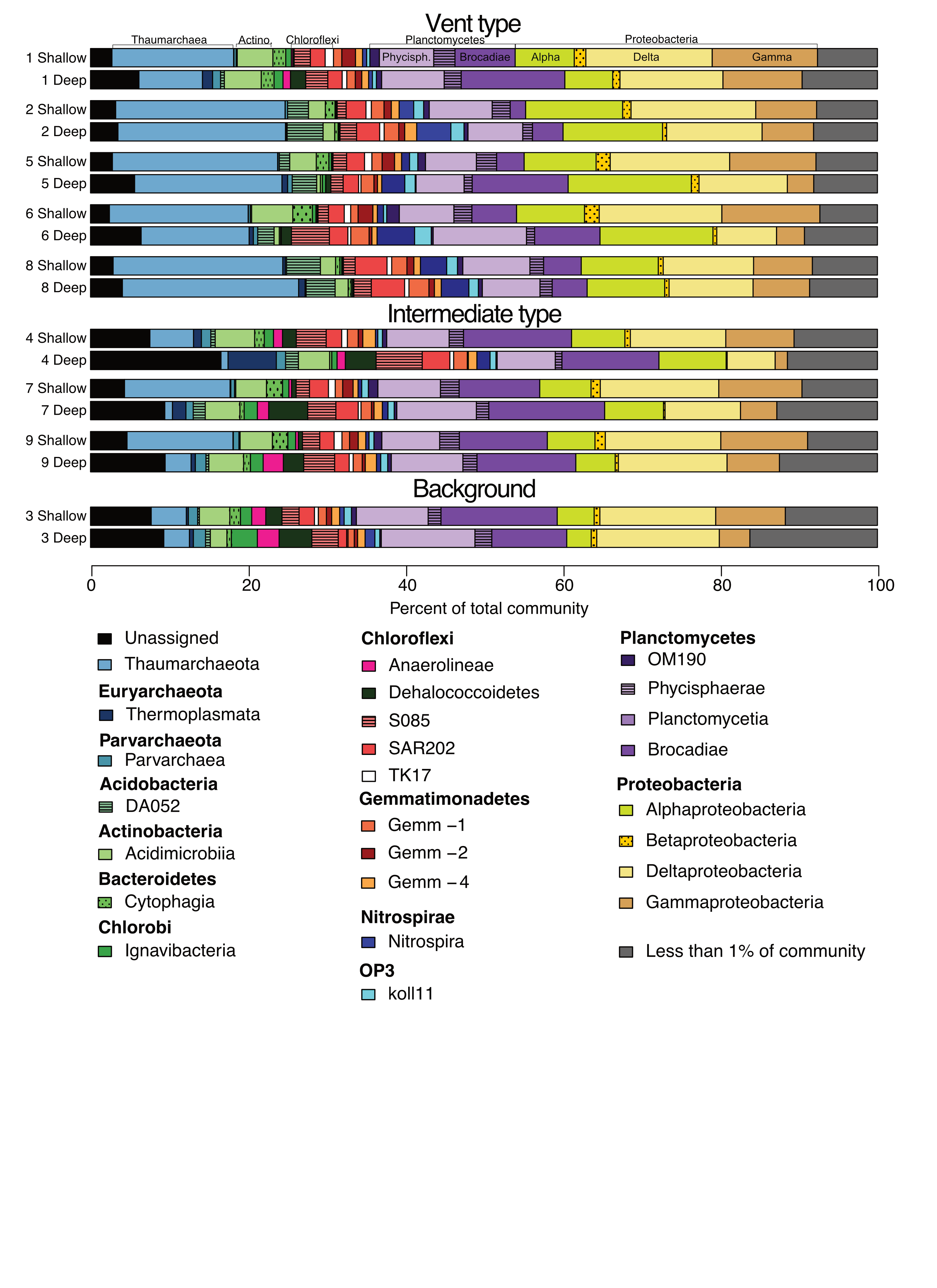

Supplement: FIGURE S4 — Class level assignments of major groups in sediments on Dorado Outcrop, based on averages of triplicate samples. Thaumarchaea, Planctomycetes, and Proteobacteria classes dominate in the hydrothermal samples. [file Image_4.TIFF]

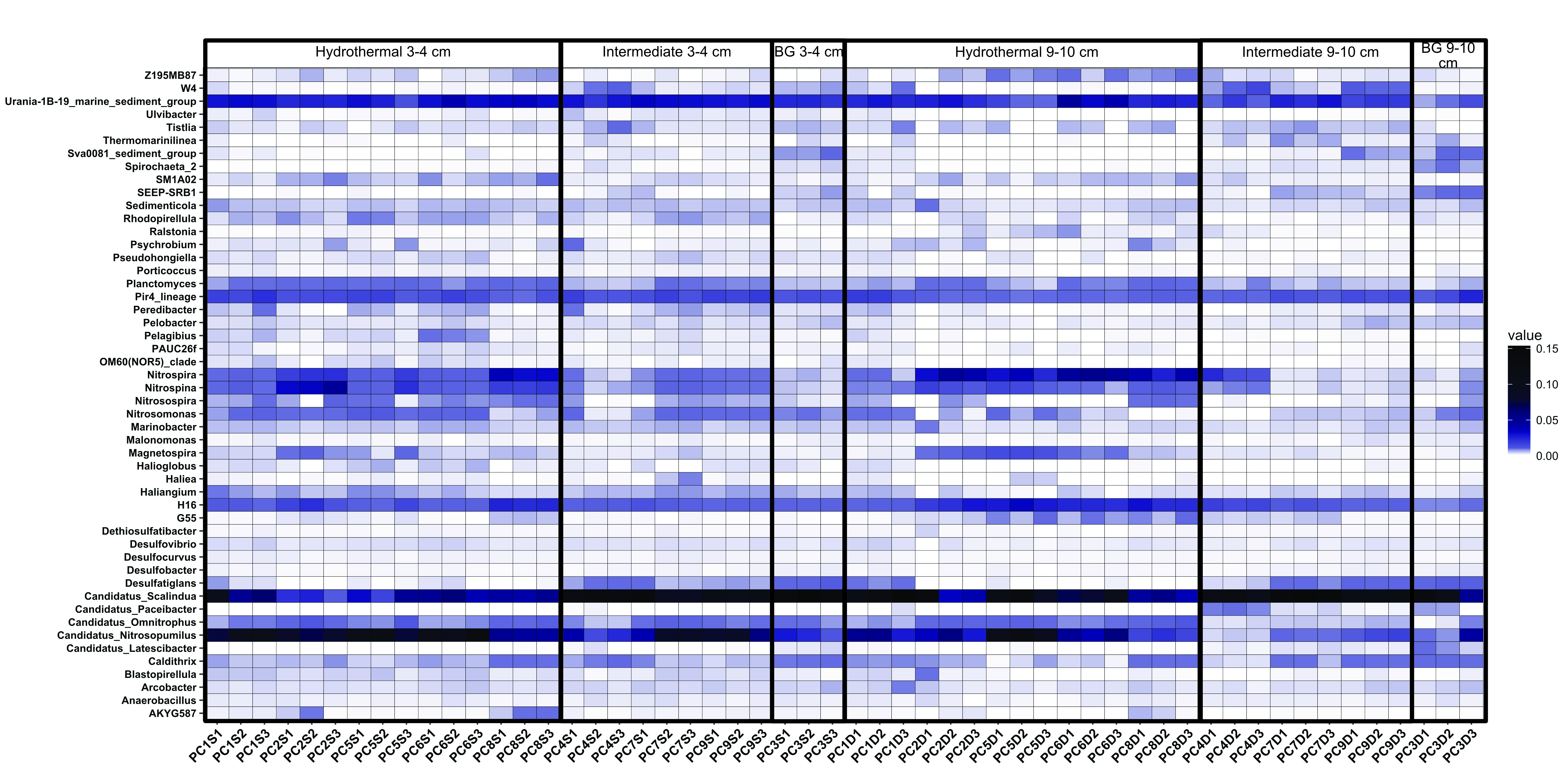

Supplement: FIGURE S5 — Relative abundances of genera at Dorado Outcrop. The x-axis includes all samples, with each replicate individually shown. The y-axis shows all assigned genera over 0.15% abundance. Candidatus Scalindua and Candidatus Nitrosopumilus are abundant in hydrothermal samples. [file Image_5.JPEG]

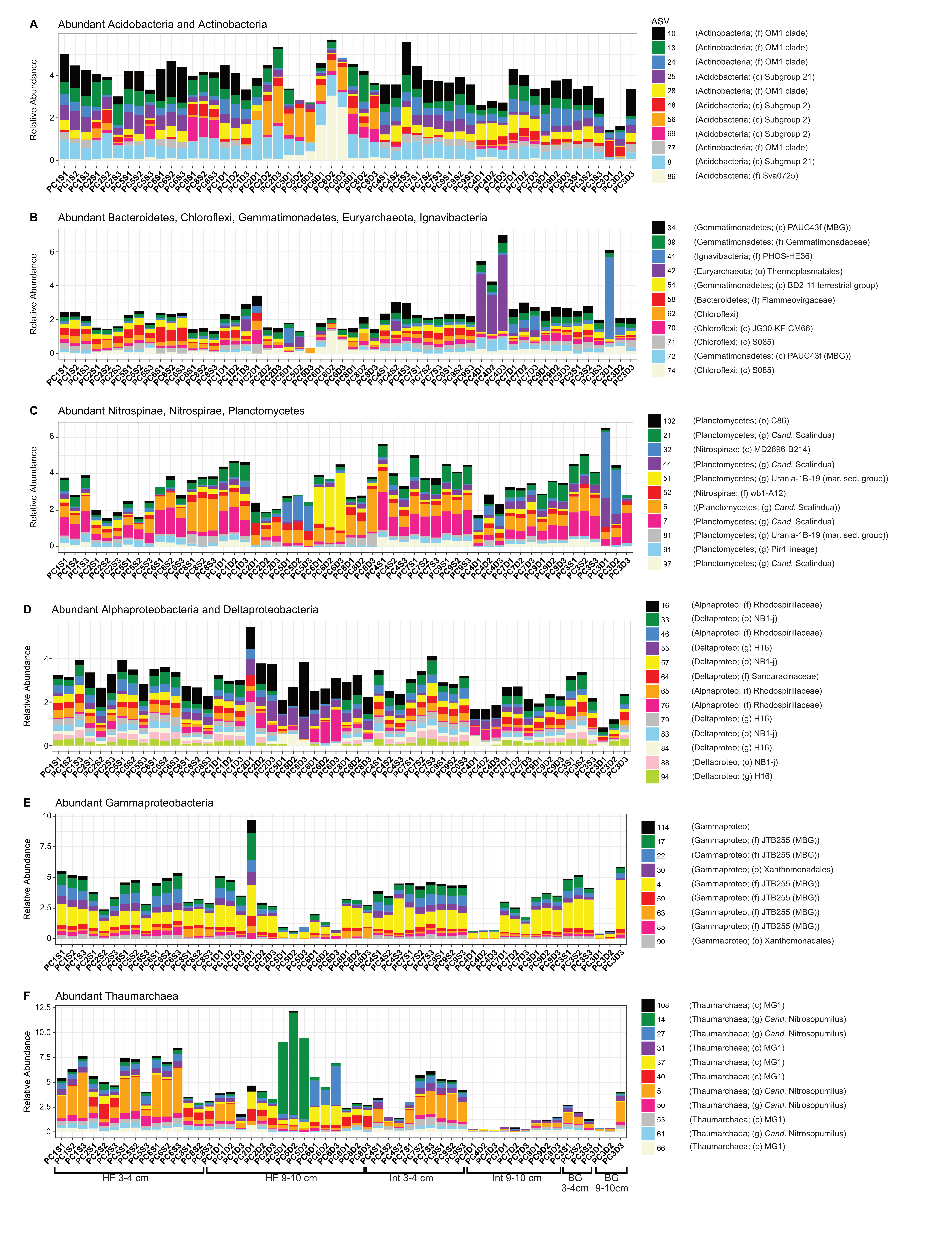

Supplement: FIGURE S7 — The abundance of the top 100 ASVs and their taxonomic assignments which showed no distinct pattern between sample types, for Acidobacteria and Actinobacteria (A); Bacteroidetes, Chloroflexi, Gemmatimonadetes, Euryarchaeota, and Ignavibacteria (B); Nitrospinae, Nitrospirae, and Planctomycetes (C); Alphaproteobacteria and Deltaproteobacteria (D); Gammaproteobacteria (E); and Thaumarchaea (F). Relative abundance is in percent of total sequences per sample. The taxonomic levels listed are phylum followed by the most specific taxonomic level assigned (c, class; o, order; f, family; g, genus) and the taxon name. ASV114 could not be assigned beyond the phylum level. [file Image_7.TIFF]

Total abundance of Top 300 OTUs, including all OTUs >1% total abundance

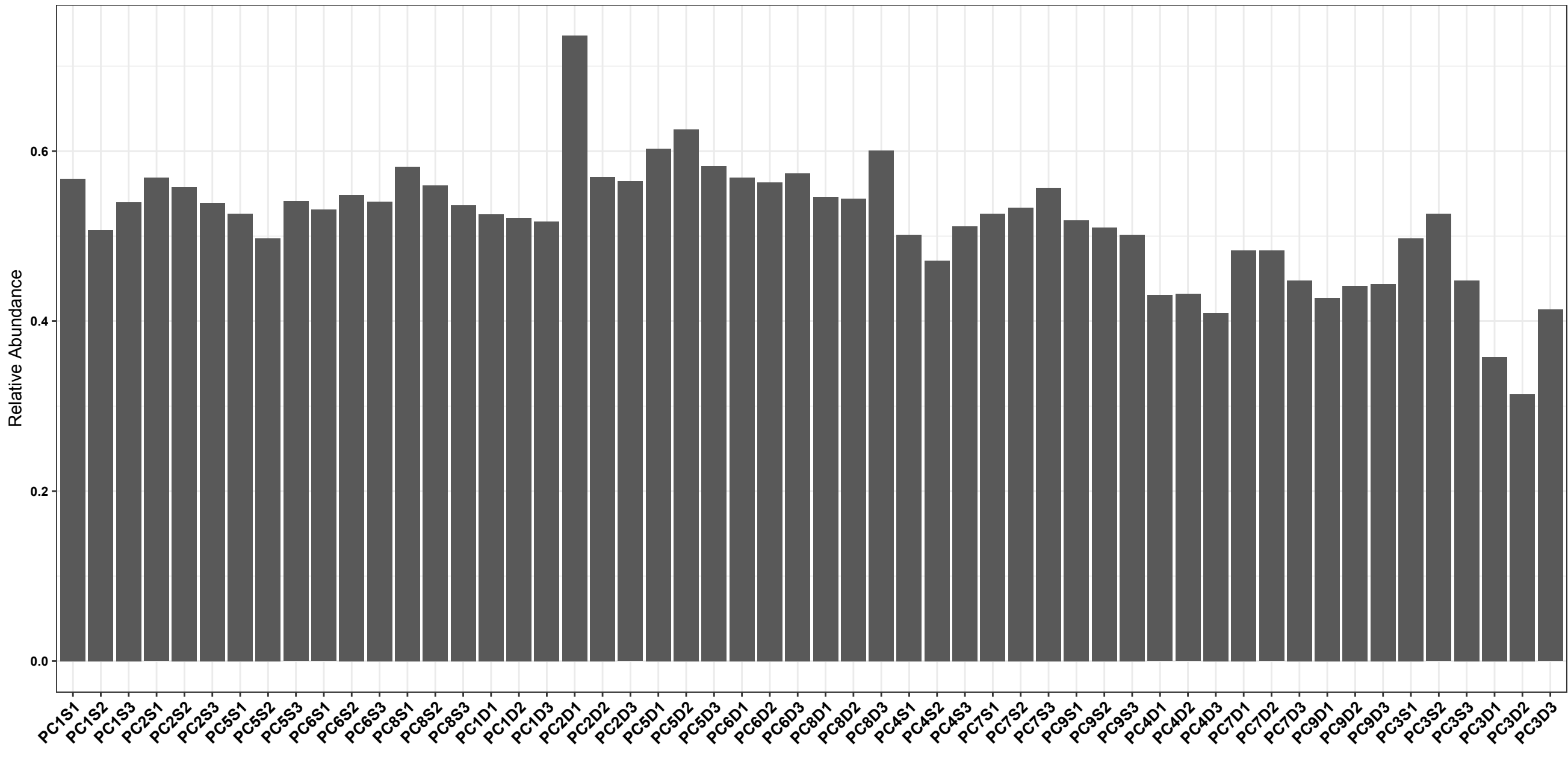

Supplement: FIGURE S8 — Combined relative abundance of the top 300 ASVs (which included all ASVs considered in Figures 4, 5) as a proportion of the total community. The top 300 ASVs represented over half of the sequences in most samples, and included all ASVs over 1% abundance. [file Image_8.PDF]

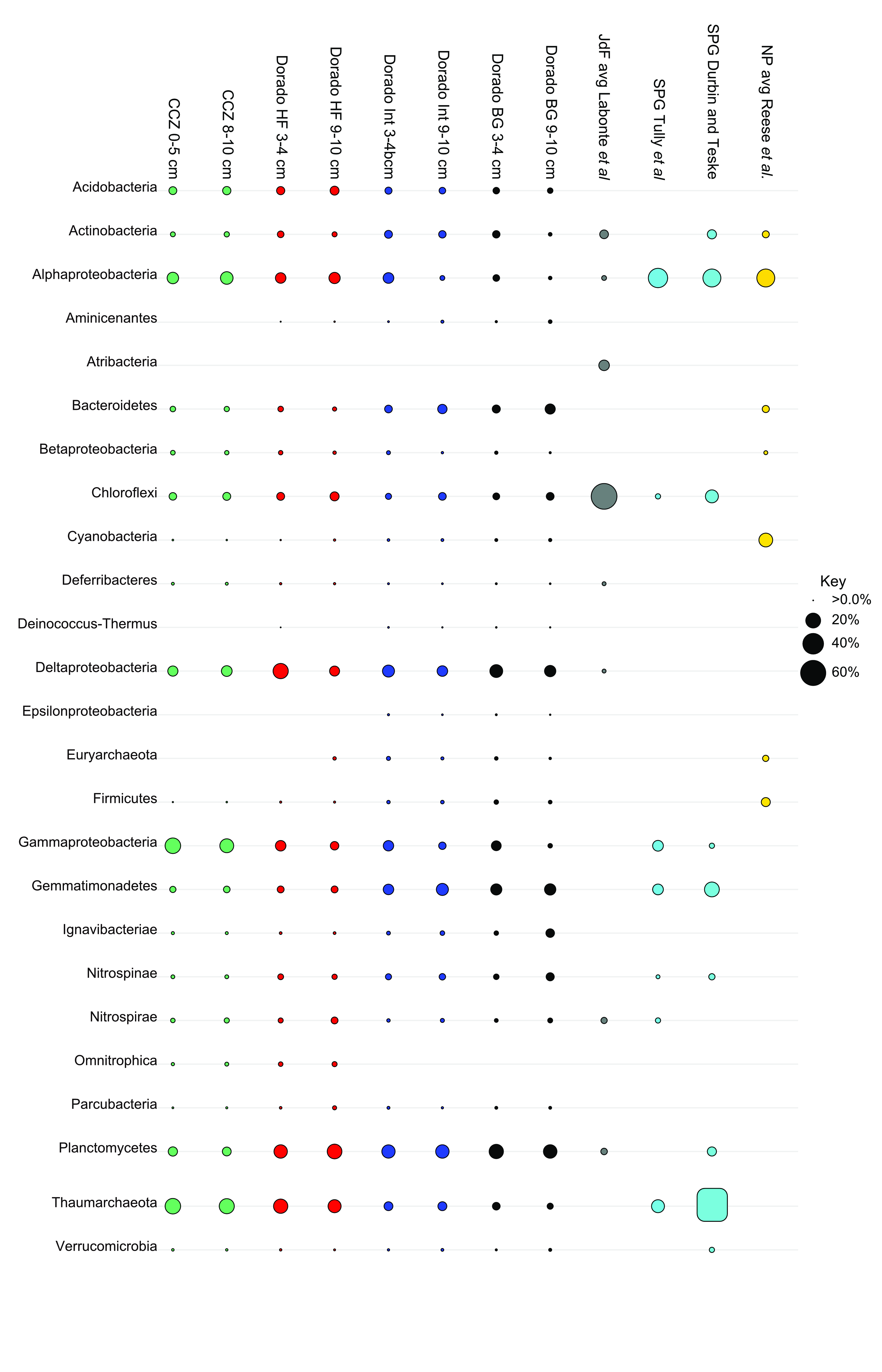

Supplement: FIGURE S9 — Comparison of Dorado Outcrop communities to communities from other deep sea sediments. Taxa are listed at the phylum level, except for the Proteobacteria, which are listed at the class level. Durbin and Teske separated Archaea and Bacteria clone library percentages, so the Thaumarchaea (MGI), which were 100% of the clones, are displayed as a large rounded square, and all Bacteria clones are percentages of only the Bacteria community. [file Image_9.JPEG]
