# Supplementary material for: Sediment Microbial Communities Influenced by Cool Hydrothermal Fluid Migration
Source: Front Microbiol. 2018 Jun 13;9:1249. doi: 10.3389/fmicb.2018.01249 (PMC6008377; doi:10.3389/fmicb.2018.01249)
Supplement: TABLE S1 — Sequence statistics during processing in DADA2. [file Table_1.docx]

**Table S1.** Sequence statistics during processing in DADA2.

| **Sample core** | **Depth (cmbsf)** | **Replicate** | **Input Sequences** | **Filtered Sequences** | **Denoised**  **Sequences** | **Non-chimeric sequences** |
| --- | --- | --- | --- | --- | --- | --- |
| PC1 | 3-4 | 1 | 69,848 | 69,848 | 69,848 | 68,979 |
|  |  | 2 | 122,247 | 122,247 | 122,247 | 117,475 |
|  |  | 3 | 51,003 | 51,003 | 51,003 | 50,543 |
|  | 9-10 | 1 | 76,350 | 76,350 | 76,350 | 75,436 |
|  |  | 2 | 88,730 | 88,730 | 88,730 | 87,101 |
|  |  | 3 | 61,471 | 61,471 | 61,471 | 60,995 |
| PC2 | 3-4 | 1 | 72,689 | 72,689 | 72,689 | 71,315 |
|  |  | 2 | 101,447 | 101,447 | 101,447 | 98,988 |
|  |  | 3 | 86,472 | 86,472 | 86,472 | 83,651 |
|  | 9-10 | 1 | 6,786 | 6,786 | 6,786 | 6,786 |
|  |  | 2 | 91,073 | 91,073 | 91,073 | 88,822 |
|  |  | 3 | 64,783 | 64,783 | 64,783 | 62,781 |
| PC3 | 3-4 | 1 | 68,230 | 68,230 | 68,230 | 67,191 |
|  |  | 2 | 54,819 | 54,819 | 54,819 | 54,231 |
|  |  | 3 | 90,734 | 90,734 | 90,734 | 88,941 |
|  | 9-10 | 1 | 90,048 | 90,048 | 90,048 | 88,860 |
|  |  | 2 | 65,571 | 65,571 | 65,571 | 65,165 |
|  |  | 3 | 82,013 | 82,013 | 82,013 | 81,057 |
| PC4 | 3-4 | 1 | 76,050 | 76,050 | 76,050 | 74,954 |
|  |  | 2 | 82,935 | 82,935 | 82,935 | 82,297 |
|  |  | 3 | 106,678 | 106,678 | 106,678 | 104,776 |
|  | 9-10 | 1 | 81,908 | 81,908 | 81,908 | 81,279 |
|  |  | 2 | 68,339 | 68,339 | 68,339 | 67,626 |
|  |  | 3 | 90,222 | 90,222 | 90,222 | 89,482 |
| PC5 | 3-4 | 1 | 84,328 | 84,328 | 84,328 | 82,828 |
|  |  | 2 | 85,920 | 85,920 | 85,920 | 83,688 |
|  |  | 3 | 90,245 | 90,245 | 90,245 | 88,814 |
|  | 9-10 | 1 | 66,030 | 66,030 | 66,030 | 65,492 |
|  |  | 2 | 69,441 | 69,441 | 69,441 | 68,132 |
|  |  | 3 | 37,399 | 37,399 | 37,399 | 37,135 |
| PC6 | 3-4 | 1 | 68,018 | 68,018 | 68,018 | 66,771 |
|  |  | 2 | 55,296 | 55,296 | 55,296 | 54,311 |
|  |  | 3 | 53,624 | 53,624 | 53,624 | 53,056 |
|  | 9-10 | 1 | 54,800 | 54,800 | 54,800 | 54,437 |
|  |  | 2 | 48,453 | 48,453 | 48,453 | 48,052 |
|  |  | 3 | 58,643 | 58,643 | 58,643 | 58,254 |
| PC7 | 3-4 | 1 | 72,420 | 72,420 | 72,420 | 71,248 |
|  |  | 2 | 65,874 | 65,874 | 65,874 | 65,010 |
|  |  | 3 | 44,776 | 44,776 | 44,776 | 44,430 |
|  | 9-10 | 1 | 71,307 | 71,307 | 71,307 | 70,953 |
|  |  | 2 | 84,376 | 84,376 | 84,376 | 83,690 |
|  |  | 3 | 82,065 | 82,065 | 82,065 | 81,583 |
| PC8 | 3-4 | 1 | 112,564 | 112,564 | 112,564 | 109,221 |
|  |  | 2 | 115,717 | 115,717 | 115,717 | 112,703 |
|  |  | 3 | 130,957 | 130,957 | 130,957 | 127,268 |
|  | 9-10 | 1 | 125,284 | 125,284 | 125,284 | 121,154 |
|  |  | 2 | 94,144 | 94,144 | 94,144 | 91,058 |
|  |  | 3 | 74,504 | 74,504 | 74,504 | 72,546 |
| PC9 | 3-4 | 1 | 100,664 | 100,664 | 100,664 | 98,327 |
|  |  | 2 | 124,901 | 124,901 | 124,901 | 121,796 |
|  |  | 3 | 104,294 | 104,294 | 104,294 | 102,494 |
|  | 9-10 | 1 | 125,769 | 125,769 | 125,769 | 123,031 |
|  |  | 2 | 118,540 | 118,540 | 118,540 | 116,313 |
|  |  | 3 | 119,385 | 119,385 | 119,385 | 117,950 |
| Extraction blanks | - | 1 | 98,527 | 90,263 | 90,263 | 50,937 |
|  |  | 2 | 73,836 | 68,294 | 68,294 | 42,542 |
|  |  | 3 | 96,769 | 88,320 | 88,320 | 55,279 |
| Total Sequences |  |  | 4,659,316 | 4,637,061 | 4,637,061 | 4,459,234 |
